# Heterologous Expression and Immunoreactivity of Wheat Amylase–Trypsin Inhibitors

**DOI:** 10.3390/ijms27198857

**Published:** 2026-10-04

**Authors:** Lisa Call, Thomas Svoboda, Andrea Krisai, Tim Hacker, Kathrin Lauter, Stefano D’Amico, Birgit Linhart, Agnès Amouric, Mickaël Lafond, Thierry Giardina, Motohiro Ebisawa, Rudolf Valenta, Heinrich Grausgruber

**Affiliations:** 1 Department of Agricultural Sciences, BOKU University, 3430 Tulln an der Donau, Austria; lisa.call@ages.at (L.C.); thomas.svoboda@boku.ac.at (T.S.); kathrin.lauter@boku.ac.at (K.L.); 2 AGES— Austrian Agency for Health and Food Safety, 1220 Vienna, Austria; stefano.damico@ages.at; 3 Institute of Pathophysiology and Allergy Research, Medical University of Vienna, 1090 Vienna, Austria; birgit.linhart@meduniwien.ac.at (B.L.); rudolf.valenta@meduniwien.ac.at (R.V.); 4 iSm2, CNRS, Aix-Marseille Université, Centrale Marseille, 13397 Marseille, France; agnes.amouric@univ-amu.fr (A.A.); michael.lafond@univ-amu.fr (M.L.); thierry.giardina@univ-amu.fr (T.G.); 5 Clinical Research Center for Allergy and Rheumatology, NHO Sagamihara National Hospital, Sagamihara 252-0392, Kanagawa, Japan; mebisawa@foodallergy.jp; 6 Center for Molecular Allergology, Karl Landsteiner University of Health Sciences, 3500 Krems, Austria

**Keywords:** amylase–trypsin inhibitors, heterologous expression system, *Pichia pastoris*, recombinant protein expression, *Triticum* sp., wheat-related disorders

## Abstract

Wheat amylase–trypsin inhibitors (ATIs) are known for their allergenic properties, but their precise role in celiac disease and other wheat-related disorders remains unclear. Pure ATI preparations provide a basis for detailed biochemical and immunologic characterization; however, the complexity of isolating ATIs in the absence of other immunoreactive wheat proteins is an ongoing challenge. To address this limitation, bacterial and yeast heterologous expression systems were evaluated for the production of pure ATI preparations. While the synthesis of recombinant ATIs 0.28 and CM3 in *Escherichia coli* was challenging regarding protein solubility, promising results were observed for ATIs 0.19, CM1 and CM3 expressed in *Pichia pastoris*, both in terms of solubility and protein yields. The biochemical and immunoreactive properties of these recombinant ATIs were evaluated by SDS-PAGE, MALDI-TOF MS, LC-MS/MS, circular dichroism analysis, and in vitro inhibitory and immunoassays, and compared to native ATIs isolated from wheat. Our findings shed light on the difficulties in isolating pure ATIs and provide valuable insights into the potential impact of these proteins on human health. By using innovative expression systems, we aim to contribute to the detailed biochemical and immunogenic characterization of ATIs and improve the understanding of their role in wheat-related disorders and associated allergic responses.

## 1. Introduction

Although wheat (*Triticum* L.) and wheat-based products represent essential pillars of the human diet, their proteins are also associated with various diseases, including autoimmune (celiac disease, CD), allergic (wheat allergy, WA; and wheat-dependent exercise-induced anaphylaxis, WDEIA), and non-autoimmune and non-allergic (non-celiac wheat sensitivity; NCWS) responses, as well as irritable bowel syndrome (IBS) [1]. While in some surveys about 10–15% of the population self-reported wheat-related disorders (WRDs), clinical confirmation is significantly lower [2,3,4,5]. Clinical manifestations of these diseases are manifold and include, among others, malnutrition, dermatitis and neurological symptoms in the case of CD, and anaphylaxis in the case of WA. Symptoms of NCWS are less specific and may overlap with other WRDs and, thus, NCWS can be diagnosed only after CD and WA have been excluded [5,6,7,8,9,10,11]. Although fructans in particular are the main triggers of IBS, causing, e.g., abdominal pain and diarrhea, it seems likely that certain wheat components also play a role in this disease [11].

While gluten proteins are recognized as dominant triggers of WRDs, other proteins, most notably amylase–trypsin inhibitors (ATIs), are gaining increasing attention as they also appear to be involved in the clinical pathogenesis of WRDs [12]. ATIs, a group of water-soluble proteins with different isoforms and molecular masses ranging from 11 kDa to 16 kDa, are crucial for the natural defense mechanism of the wheat plant by suppressing the digestive enzymes of invading pests and parasites [13,14]. All ATI isoforms are predicted to have an overall structure of four α-helices stabilized by 4–5 disulfide bonds [15]. Although the amino acid sequences of some ATI isoforms contain potential glycosylation sites, there is only information available for one ATI subunit (i.e., CM16) that it exists both as a glycosylated and non-glycosylated form [16]. While gluten proteins are known to be responsible for CD and WA, no clear scientific evidence is yet available on the role of ATIs in WRDs [10,11]. Recent studies suggest that ATIs are not only the main triggers of baker’s asthma, a respiratory form of WA, but are also involved in the pathogenesis of NCWS by activating the production of inflammatory cytokines and chemokines [17,18] and CD, as immune responses to ATIs have been detected in serum samples from CD patients [19,20]. Despite emerging evidence of the involvement of ATIs in WRDs, their immunogenic properties and role in CD and NCWS remain poorly understood. One of the major challenges in studying ATIs and their potential to trigger adverse reactions is the lack of pure ATI preparations that can be used for testing. Due to the structural similarity of ATIs to some gluten proteins and other proteins belonging to the so-called prolamin superfamily [21], the isolation of pure ATIs in the absence of other immunogenic proteins and crucial allergens is difficult to accomplish, albeit essential for adequate investigation of their antigenic and allergenic properties and definitive proof of their involvement in CD and NCWS.

The heterologous expression of ATIs in suitable expression systems is being explored as an alternative approach to circumvent the complexities of isolation. Although the use of *Escherichia coli* as a cell factory is well established, difficulties in using *E. coli* as a platform for the expression of ATIs have been reported [22]. Given the complex structures of ATIs containing multiple disulfide bonds, their expression might be favored in eukaryotic hosts (e.g., the yeast expression system *Pichia pastoris*, now classified as *Komagataella phaffii*), which are generally better suited to perform required post-translational modifications (PTMs) [23]. However, the glycosylation of proteins, which is likely to occur in yeast expression systems, may affect their immunogenic properties [24,25]. Therefore, the selection of an appropriate *P. pastoris* strain is essential to avoid the accumulation of long glycosyl chains.

This study presents a comprehensive purification procedure for ATIs with a series of subsequent analytical methods to verify the final purity of the protein preparation. By comparing the heterologous expression of ATIs in *E. coli* (isoforms 0.28 and CM3) and *P. pastoris* (isoforms 0.19, CM1, and CM3), we seek to provide an effective alternative strategy to generate pure ATIs devoid of other immunoreactive contaminants. Through these efforts, we aim to elucidate the role of ATIs in WRDs and advance our understanding of their impact on human health. Among the 19 ATI isoforms previously described [26], the selection for heterologous expression in this study is based on their presumed function as strong activators of the immune system [17,20] and their prevalence in wheat [27].

## 2. Results

### 2.1. Purification of ATIs from Natural Source

**Figure 1:**
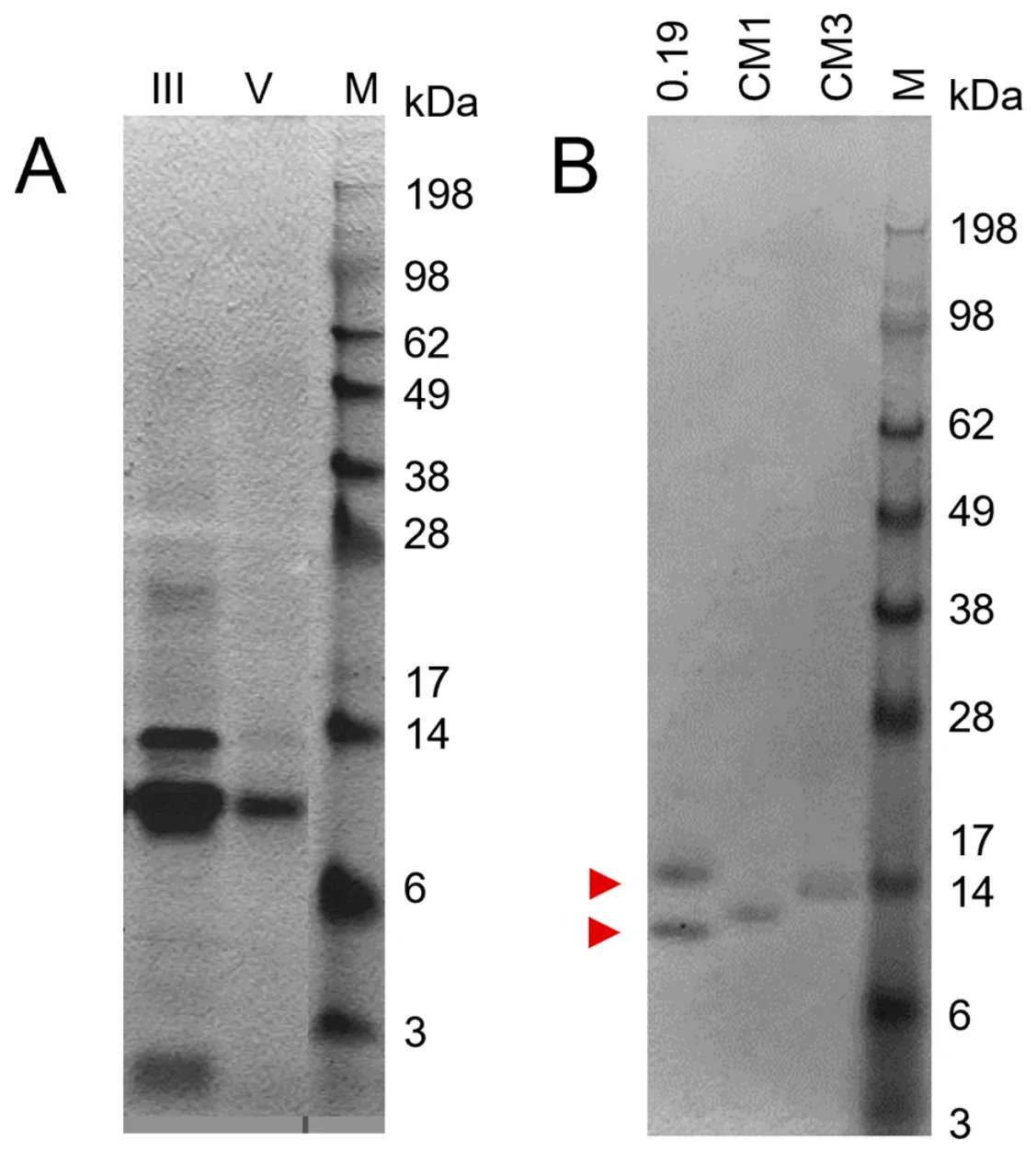
Electrophoretic evaluation of the composition of natural and recombinant ATIs: (**A**) natural ATI preparation (nATI) at different stages of the purification process (stage III and stage V); (**B**) purified recombinant ATIs (rATIs) 0.19, CM1, and CM3 expressed in *P. pastoris*. Red arrows highlight the two bands obtained for ATI 0.19. Lane M comprises marker polypeptides with the following molecular weights: 198, 98, 62, 49, 38, 28, 17, 14, 6, 3 kDa (InvitrogenTM SeeBlueTM Plus2 Pre-Stained Protein Standard).

The purification of ATIs from wheat was carried out using a multi-stage purification process to obtain an ATI preparation with high purity. Figure 1A shows the composition of the nATI preparation at two different stages of the purification process. Lane III shows the nATI preparation after CM and PBS purification (stage III), while lane V shows the final preparation at the end of the entire purification procedure (stage V). In both lanes, protein bands with masses of about 10–15 kDa were highly abundant, corresponding to the molecular weights of ATIs. However, the bands of lower and higher masses clearly visible in lane III indicate the high number of impurities present in the ATI preparation at this stage. As for lane V, which represents the final preparation at the end of the complete clean-up process, no impurities were detected by SDS-PAGE.

**Figure 2:**
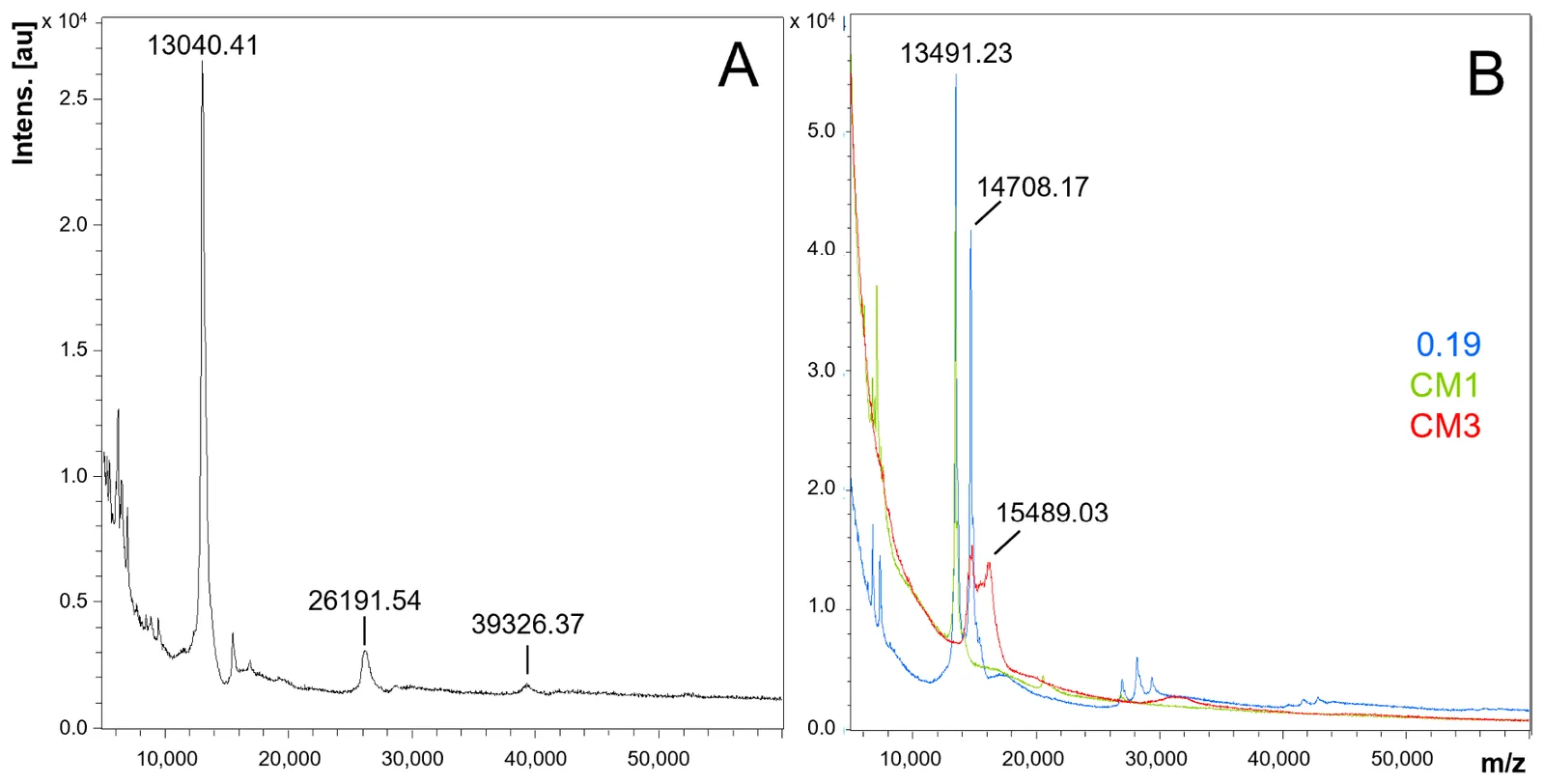
MALDI-TOF MS spectra (5000–60,000 Da) of natural and recombinant ATIs: (**A**) nATI preparation after purification; (**B**) rATIs 0.19, CM1 and CM3 from *Pichia pastoris* after purification.

MALDI-TOF MS analysis (Figure 2A) of the final nATI preparation was performed to verify the SDS-PAGE results. The spectrum obtained showed a large abundance of proteins at around 13 kDa (dimers at around 26 kDa and trimers at around 39 kDa) and a smaller peak around 15.5 kDa, which can be assigned to major ATIs and CM3. No other distinct peaks were detected except a low abundance of proteins < 10 kDa. Thus, the MALDI-TOF MS results support the high degree of purity determined by SDS-PAGE, which only showed bands typical for the mentioned inhibitor proteins found in wheat.

In order to identify potential impurities present in the nATI preparation that may not be detectable by SDS-PAGE and MALDI-TOF MS, LC-MS/MS analysis was additionally performed. The raw data were processed using PLGS and matched with the SwissProt database. A list of identified proteins can be found in Table 1 and includes ATIs 0.19, 0.53, CM3, 0.28, and CM16. Mass spectrometric data for ATI 0.19 revealed the presence of the peptide LQCNGSQVPEAVLR in duplicate with a mass difference of 726, which corresponds to the mass of a GlcNAcHex2 group, indicating that ATI 0.19 is partially glycosylated. In addition to the detection of ATIs, LC-MS/MS analysis also revealed the potential presence of non-specific lipid transfer proteins (nsLTPs), thioredoxin, and avenin-like proteins (ALPs). However, it is important to note that their presence in the nATI preparation could not be unequivocally confirmed, as only one unique peptide was identified for each protein. The high scores for ATIs and the distinctly lower ones for the other proteins are in agreement with the relatively high purity of the nATI preparation.

Finally, the presence of gluten proteins and other wheat allergens in the nATI preparation was tested by ELISA. For this purpose, rabbit antisera containing polyclonal antibodies to specific wheat allergens were used, including profilin (Tri a 12), nsLTP (Tri a 14), beta-amylase (Tri a 17), gamma-gliadin (Tri a 20), thioredoxin (Tri a 25), high-molecular-weight glutenin (HMW, Tri a 26), 1-cys-peroxiredoxin (Tri a 32), dehydrin (Tri a 35), low-molecular-weight glutenin (LMW, Tri a 36), alpha-purothionin (Tri a 37), serine protease inhibitor-like protein (Tri a 39), and ALP. This technique allows for the highly sensitive detection of low levels of those wheat proteins in the purified nATI preparation. The results of the immunoassays are shown in Supplementary Figure S1 and indicate a high degree of purity in the final nATI preparation (stage V).

### 2.2. Heterologous Expression of ATIs in E. coli and P. pastoris

The synthesis of recombinant ATIs was performed to circumvent the difficulties in ATI purification from natural sources. First, the expression of ATIs 0.28 and CM3 was performed in *E. coli*. After expression, proteins in the soluble fraction (PBS) and in the insoluble fraction (re-solubilized in 8 M urea) were visualized by SDS-PAGE to evaluate the solubility of the target ATI proteins. Protein bands corresponding to ATIs 0.28 and CM3 were found in the insoluble protein fraction (P) (Supplementary Figure S2A). To allow the proteins to refold and recover their native state, a three-buffer system was used for affinity purification. The proteins were loaded onto the HisTrap^TM^ FF crude column under denaturing conditions. Eventually, the buffer was slowly changed to 0.05 M PBS (pH 7.8) containing 0.15 M NaCl and 0.005 M imidazole over 22.5 column volumes to allow refolding of the proteins to a native state. Finally, proteins were eluted by gradual buffer exchange to 0.05 M PBS (pH 7.8) containing 0.15 M NaCl and 0.5 M imidazole, and fractions of 1.5 mL were collected and pooled after SDS-PAGE analysis. The purified proteins were concentrated using Amicon Ultra-15 spin columns with a cut-off of 10 kDa (Merck), while at the same time, the buffer was exchanged for 0.05 M PBS (pH 7.8) containing 0.2 M NaCl to remove imidazole. Although the presence and purity of the His6-tagged ATI proteins were confirmed by SDS-PAGE (Supplementary Figure S2B) and Western blot using 6× His tag antibodies (Invitrogen), anti-rabbit secondary antibodies (Active Motif, Inc., Carlsbad, US) and Clarity Western ECL substrate (Bio-Rad), the proteins began to precipitate again soon after purification. Therefore, ATIs 0.28 and CM3 expressed in *E. coli* were not further characterized in this study.

As disulfide bond formation and, thus, protein folding is favored in eukaryotic hosts, a *P. pastoris* expression system was selected for the synthesis of recombinant ATIs 0.19, CM1, and CM3. The production of the target proteins in *P. pastoris* at different time intervals during three days of heterologous expression is shown in Supplementary Figure S3. The protein bands at around 15 kDa correspond to the target ATI proteins. While the protein yield of ATIs without His6 tag (W/O His tag) increased steadily over time, the gels showed only weak bands for ATIs with His6 tag (W His tag) even on the final day of expression, but a greater number of impurities. Given the presence of impurities even after affinity purification and the low yield of target proteins, His6-tagged ATI proteins were not used for further purification or analysis in our study. The purity of protein preparations containing ATIs without His6 tag was verified by SDS-PAGE after heterologous expression and purification by IEX and SEC, and was >95% (Figure 1B). A single major protein band was found for CM1 and CM3 with apparent molecular weights of around 13 kDa and 15 kDa, respectively. ATI 0.19, on the other hand, showed two major bands between 12 kDa and 15 kDa.

**Figure 3:**
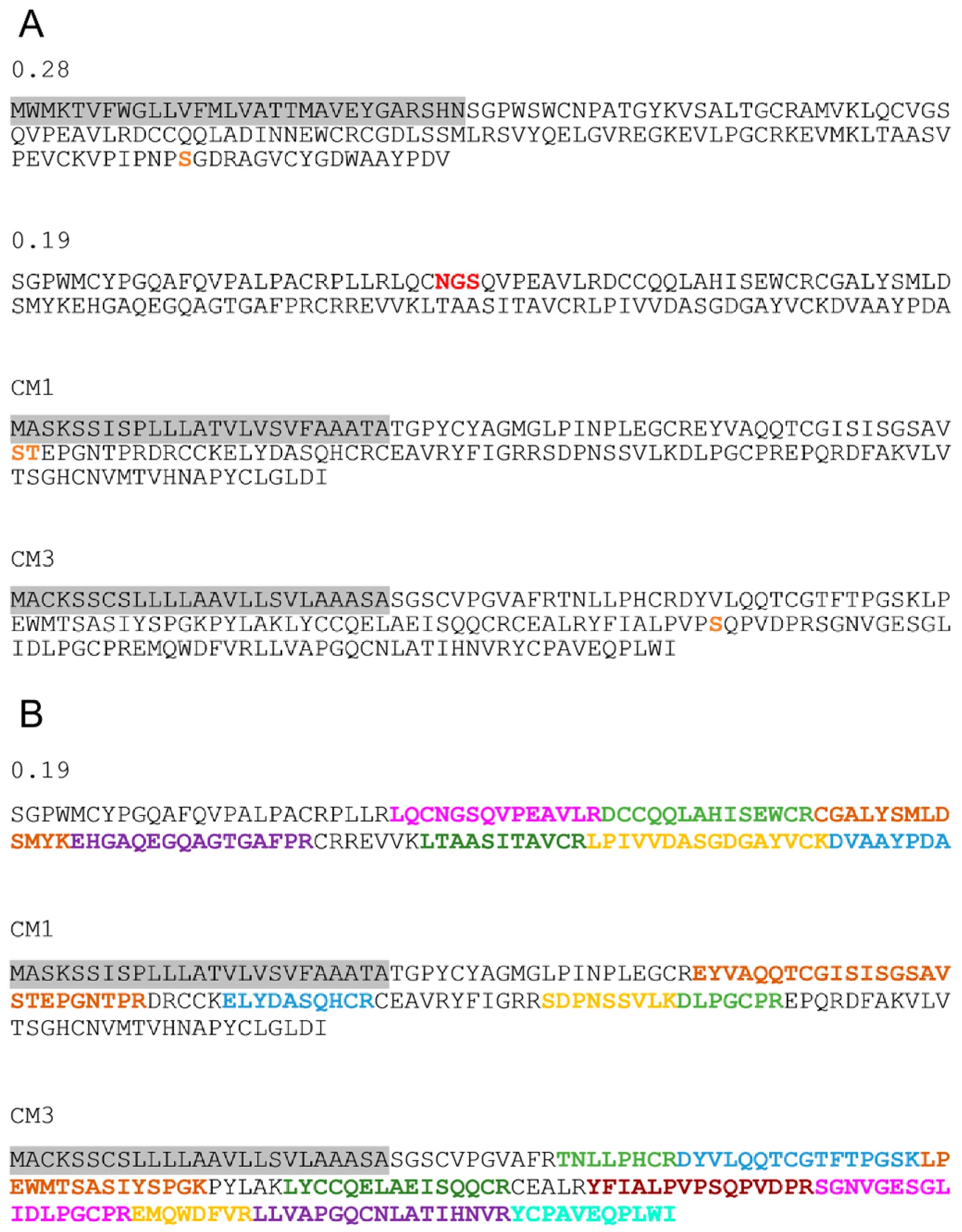
Amino acid sequences of target ATI proteins for heterologous expression in *Escherichia coli* and *Pichia pastoris*. N-terminal signal peptides are highlighted in gray, while areas without highlighting represent the mature forms of the proteins. (**A**) Amino acids shown in orange and red indicate potential O- and N-glycosylation sites, respectively. (**B**) Amino acid sequences highlighted in color indicate peptides detected by LC-MS/MS after tryptic digestion.

The MALDI-TOF MS results (Figure 2B) for the recombinant ATI 0.19 confirmed the presence of two main protein fractions with masses of 13,491 Da and 14,708 Da, respectively. The two peaks, as well as the two bands detected on SDS-PAGE gels, indicate that part of the protein preparation is in an N-glycosylated form as N-glycosylation was already predicted in silico for ATI 0.19 (Figure 3A). The mass difference of 1217 Da suggests the attachment of an N-linked N-acetyl-D-glucosamine, probably with five mannosyl residues (e.g., N-GlcNAcMan5 1234 Da; N-linked residue 1216 Da), as engineered for the *P. pastoris* strain SuperMan5. MALDI-TOF MS spectra for ATIs CM1 and CM3 confirmed the presence of proteins with masses of 13,441 Da and 15,489 Da, respectively. Protein identities were confirmed after tryptic digestion and subsequent LC-MS/MS analysis, yielding sequence coverages of 74% (score: 1133, peptides: 8), 43% (score: 156, sequences: 5), and 62% (score: 204, sequences: 6) for 0.19, CM1, and CM3, respectively (Figure 3B). For rATI 0.19, the peptide LQCNGSQVPEAVLR, which contains the potential N-glycosylation site (Figure 3A), was found with two different masses, supporting the assumption that this protein is present in glycosylated and non-glycosylated forms. The mass difference is consistent with a linked N-GlcNAcMan5, but was not verified with additional measurements after the release of the glycosylation site (e.g., by LC-MS/MS of the glycopeptide after PNGase F treatment).

Estimates of secondary structure composition through circular dichroism analysis (CDA) revealed a predominantly disordered structure of the recombinant proteins (41%), with strand segments comprising 36% of the composition.

### 2.3. Immunoreactivity of Natural and Recombinant ATIs

**Figure 4:**
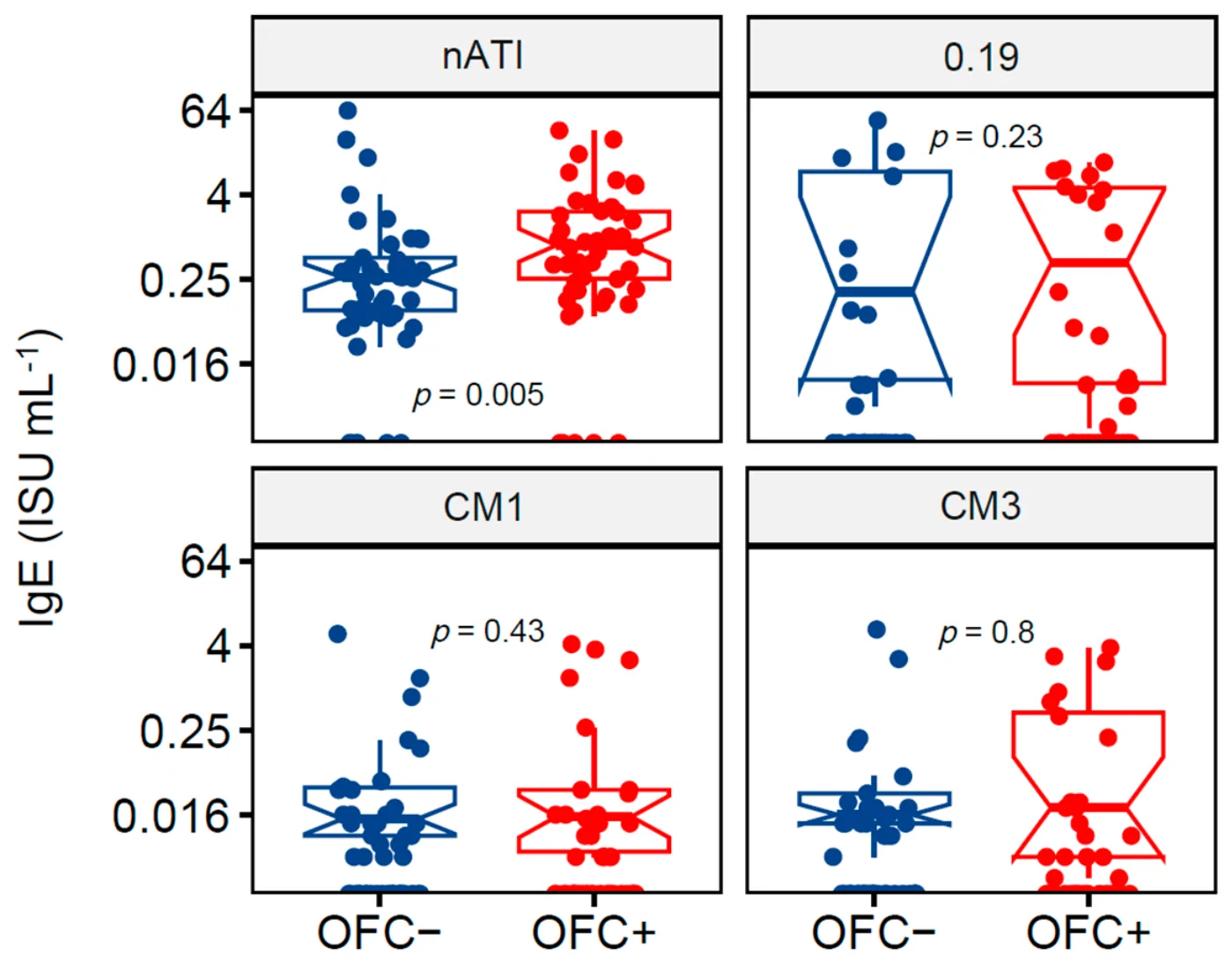
IgE reactivity to natural wheat ATIs (nATI) and recombinant ATIs 0.19, CM1 and CM3 expressed in *Pichia pastoris* in a cohort of Japanese children phenotyped having a positive (OFC+; *n* = 47) or negative oral food challenge (OFC−; *n* = 47) with wheat. Indicated levels of significance correspond to the Wilcoxon signed-rank test between to two groups of patients; y-axes of subplots are displayed in a base-2 logarithmic scale.

The ATI preparations from this study (nATI, rATIs 0.19, CM1, and CM3 from *P. pastoris*) were further subjected to antigenic characterization in an in vitro study using microarray chip technology. The results are summarized in Figure 4, as well as in Supplementary Tables S1 and S2, and confirm the IgE-binding activity of wheat ATIs, as a total of 67 patients (71.3%) showed a positive IgE reactivity (>0.1 ISU mL^−1^) to nATIs and 24 patients (25.5%) to at least one rATI, with the highest frequency for rATI 0.19 (*n* = 16, 17.0%), followed by CM3 (*n* = 11, 11.7%) and CM1 (*n* = 10, 10.6%). While IgE reactivity to nATIs was more frequent, higher IgE levels in responsive subjects were found for rATIs (019: $\tilde{x}$ = 6.4 ISU; CM1: $\tilde{x}$ = 1.4 ISU; CM3: $\tilde{x}$ = 0.9 ISU) compared to nATIs ($\tilde{x}$ = 0.62 ISU) (Supplementary Table S1). The difference of IgE reactivity between the group of wheat allergic children with (OFC+) and without a positive oral food challenge (OFC−) was significant (*p* = 0.005) only for the nATI preparation (Figure 4; Supplementary Table S2). The IgE-binding activity of the nATI protein on the commercial ImmunoCAP ISAC E112i array was similar and showed a correlation of *r* = 0.95 (*p* < 0.001) to the IgE-binding activity of our nATI preparation. The significance between the OFC+ and OFC− groups, at *p* < 0.001, was slightly more pronounced than for our nATI preparation (Supplementary Tables S1 and S2).

## 3. Discussion

The involvement of wheat ATIs in CD and NCWS is still debated on account of the challenges in studying their immunoreactive properties. There is also uncertainty about the allergenic potential of individual ATI isoforms with respect to allergy. It is widely recognized that gluten preparations that have been considered pure and used in vitro and in clinical studies of adverse reactions to gluten also contain significant amounts of other protein components, including ATIs. Moreover, isolated ATI preparations used to study their in vitro immunoreactivity also contain unidentified proteins [28] that could have a significant impact on the results, or their purity has not been verified. Consequently, until pure ATI preparations with known composition are available, the fact that these components may also contribute to the observed effects cannot be excluded.

SDS-PAGE of the nATI preparation revealed highly abundant protein bands with masses of about 10–15 kDa, which correspond to the molecular weights of ATIs. Thus, the two-step purification procedure used can be considered effective in producing a fraction enriched in ATIs, although significant losses of ATIs could be observed after each purification step. Impurities still present after purification stage III demonstrate the necessity for a multiple step purification procedure that can produce a preparation that is as pure as possible. Although the LC-MS/MS results did not provide conclusive evidence regarding the absolute purity of the final nATI preparation, they did not indicate the presence of highly immunoreactive gluten proteins, which is crucial when using nATI preparations for CD-related testing. Nevertheless, it is essential to recognize that the absence of gluten-derived peptides per se does not guarantee their complete absence in the nATI preparation, as gluten proteins are highly resistant to tryptic digestion [29]. In addition to the detection of ATIs, LC-MS/MS analysis also revealed the potential presence of nsLTP, thioredoxin, and ALP in the nATI preparation, all of which are known to be immunoreactive wheat proteins [12]. Although the signals observed for ATI preparation stage III were weaker when compared to the positive controls (recombinant proteins), this experiment provides evidence that the selective purification of wheat ATIs does not yield a pure ATI preparation. Because even minimal concentrations of wheat proteins can be expected to cause adverse reactions, the nATI preparation stage III is not suitable for investigating the involvement of ATIs in the pathogenesis of WA, CD, and other WRDs. Only an elaborate process comprising multiple purification steps based on the biochemical properties of ATIs resulted in a degree of purity sufficient for clinical studies (stage V). However, this procedure proved to be both time-consuming and resource-intensive, in addition to the overall modest ATI yields. Several authors have emphasized that ATI-enriched preparations with traces of gluten proteins, other immunoreactive albumins and globulins, or endotoxins (e.g., lipopolysaccharide) hamper clear conclusions on the role of ATIs in WRDs in both in vitro and in vivo studies [17,30,31,32].

In this context, the ability to produce specific ATI protein preparations with high purity and known composition is fundamental to elucidate their contribution to the adverse reactions observed in vitro and in vivo. In recent years, considerable progress has been made in identifying individual allergens that trigger WA. The use of purified allergen components could improve in vitro diagnostics and enable individual sensitization profiles to be linked to different clinical manifestations of WA. A nATI preparation (nTri a aA_TI) is, for example, available on the commercial ImmunoCAP ISAC E112i array. Similar absolute ISU values and the high correlation between the IgE-binding activity of our nATI preparation and the one on the commercial array demonstrates an equivalent purity of both nATI preparations. However, very few studies have addressed the heterologous expression of recombinant ATI proteins.

The first report on the expression of ATIs 0.19 and CM17 in *E. coli* demonstrated their antigenicity (IgE-binding activity) using Western blot analysis [33]. In the present study, the first attempt of expression of ATIs 0.28 and CM3 was performed in *E. coli.* Protein bands corresponding to 0.28 and CM3 were found in the insoluble protein fraction, providing a possible explanation as to why they were not found by Tundo et al. [22], who reported difficulties in expressing wheat ATIs in *E. coli*. Although the presence and purity of the ATI proteins were confirmed, the proteins began to precipitate again soon after purification, which made it difficult to spot them on the microarray chip. In contrast, García-Maroto et al. [34] and Okuda et al. [35] reported the recombinant expression of 0.28 and 0.19, respectively, in *E. coli,* but the latter authors also reported the accumulation of the protein as inclusion bodies in the insoluble fraction. Hence, the *E. coli* expression systems may be unsatisfactory to produce recombinant ATIs for further use in immunoassays.

Using the *P. pastoris* expression system for the synthesis of recombinant ATIs 0.19, CM1, and CM3 revealed protein bands at around 15 kDa corresponding to the target ATI proteins. However, the presence of a His6 tag negatively affected ATI expression, which is consistent with the study of Woestenenk et al. [36] and thus justifies the decision not to use His6-tagged ATI proteins further in our study. Furthermore, the recombinant ATIs expressed in *P. pastoris* showed a predominantly disordered structure in the CDA. Although the overall structure of four α-helices is predicted for ATIs—the structure for 0.19 was confirmed by crystallization and X-ray analysis [37]—only about 4% helical segments were estimated in the structure of the recombinant ATI proteins. These results strongly suggest that the recombinant proteins lack proper folding, affecting their structural integrity, functionality, and likely their immunoreactivity. It is plausible that other wheat proteins found in the nATI preparation, such as nsLTP, thioredoxin and ALP, may act as molecular chaperones assisting in disulfide bond formation and protein conformational folding [38]. This chaperone function has been previously described for certain plant thioredoxins [39,40], suggesting that their absence could potentially impede the correct folding of the recombinant proteins. As a further consequence, the recombinant ATI proteins exhibited no inhibitory activity against α-amylase or trypsin, highlighting the critical role of 3D protein structure in their functionality.

Similarly to the present study, Tundo et al. [22] succeeded in expressing ATIs 0.28, CM3, and CM16 in *P. pastoris* after failed attempts in *E. coli*. Moreover, the authors proved the allergenic activity of these recombinant ATIs, as they were able to induce the degranulation of basophils sensitized with IgE from wheat-allergic individuals. However, a wide range was observed in the degranulation levels of the individual proteins, suggesting significant differences due to either individual biochemical properties of the isoforms or fractions of unfolded proteins possibly present in the expressed samples, as was the case in our study.

IgE reactivity to the natural and recombinant ATIs detected by the use of an allergen microarray confirmed the high immunoreactivity of ATIs, particularly 0.19 and CM3, as reported by Junker et al. [17]. However, the findings of Junker et al. [17] refer to innate immune responses and the activation of monocytes, macrophages and dendritic cells in vitro via the TLR4-MD2-CD14 complex and may not necessarily be comparable to the antigenicity studied here. It should be mentioned that most positive responses were detected for rATI 0.19, which was found to be at least partially glycosylated. However, based on the available evidence, the attachment of five mannose residues is unlikely to substantially affect the antigenic potential of the proteins.

In general, nATIs were more frequently recognized by IgE than rATIs (i.e., not all patients with positive IgE reactivity to nATI also show positive reactivity to rATIs), suggesting that the panel of rATIs included in this study does not cover all relevant immunoreactive wheat ATIs. Furthermore, contamination of the nATI preparation by other immunoreactive proteins, as presumed by the LC-MS/MS and ELISA results, might have contributed to the higher number of IgE reactivities to nATI. The higher IgE levels found for rATIs in responsive subjects are presumably due to the purified single-protein preparations. Compared with other wheat allergens tested in the microarray, higher or similar numbers of IgE-positive sera were only found for the active form of *β*-amylase (Tri a 17, *n* = 28, 29.8%, $\tilde{x}$ = 0.2 ISU), for the 33-mer peptide originating from *α*-gliadin (*n* = 67, 71.3%, $\tilde{x}$ = 0.9 ISU), and for γ-gliadin (Tri a 20, *n* = 83, 88.3%, $\tilde{x}$ = 1.4 ISU) (Krisai et al., in preparation), highlighting a considerable role of ATIs in wheat allergy. The fact that rATIs in this study were recognized by IgE despite lacking a 3D structure suggests that their antigenicity is based on linear (i.e., sequential) epitopes, as is known, for example, for class I food allergens [41], whereas their ability to activate the pro-inflammatory receptor TLR4 was found to be based on structural confirmation [17]. Although nATIs were generally recognized more frequently by IgE than rATIs, three patients showed a positive IgE reaction to rATIs, but did not respond to nATIs. This could be attributed to the linear structure of the rATIs in this study, possibly exposing additional linear epitopes that may be relevant for certain wheat-sensitive patients.

The results of the in vitro study performed by Sánchez et al. [20] using rATI 0.19 (expression details not published) showed increased IgA and IgG antibodies (isotypes associated with active CD) in patients with CD compared to healthy controls, suggesting the involvement of wheat ATIs in the pathomechanism of CD. In contrast to the present study, the 3D structure and the functionality of the recombinant ATIs in terms of inhibitory activity were not determined in the aforementioned studies, making comparisons difficult. A recent study, however, has shown that recombinant CM3 proteins (expressed in LentiX 293T cells) are unable to inhibit α-amylase or trypsin activities [42], which was also found for the recombinant ATI proteins generated in our study. Although structural analyses were not performed for the recombinant CM3 protein, it showed α-glucosidase inhibitory activity in vitro, indicating that the protein folded correctly.

Despite the difficulties and expense associated with the production of functional recombinant ATI proteins, this study highlights the need for single ATI preparations of high purity to (i) elucidate their immunoreactive properties using complementary techniques and functional assays, and (ii) aid in diagnosis by distinguishing between sensitization, allergy, and wheat seropositivity due to cross-reactivity with other allergens. The availability of well-characterized single ATI preparations could help disentangle ATI-specific immune responses from those caused by other wheat proteins. Future studies with specific rATIs may combine in silico prediction of MHC-II-restricted T-cell epitopes with peptide-based T-cell assays and T–B cell co-culture experiments in order to identify functional T-cell epitopes [43,44] within individual ATI sequences while minimizing the confounding effects of protein folding, structure, or aggregation. Stimulation with selected ATI peptides could be used to assess T-cell activation and cytokine responses and determine whether these responses provide help to ATI-specific B cells. In this way, complementary insights into the mechanisms underlying the immunogenicity of individual ATIs could be gained.

Recombinant ATIs can be further used to produce monoclonal or polyclonal antibodies that can be used to detect and quantify ATIs in different wheat-based foods or different wheat genotypes. Similarly to the immunoassay strategy used in this study, anti-ATI antibodies can be used to detect the presence of ATIs in gluten preparations used for CD testing and exclude their contribution to the observed effects.

## 4. Materials and Methods

### 4.1. Preparation and Purification of ATIs from a Natural Source

The selective purification of native ATIs (nATIs) from wheat grains is based on their biochemical properties, including their solubility in saline solutions and chloroform/methanol (CM) mixtures, their molecular size, and their affinity for trypsin. The raw material used for preparation was a blend of common wheat (*Triticum aestivum*) cv. ‘Arnold’ from a multi-environment trial conducted in Austria from 2015 to 2017. Grains were milled into wholegrain flour in a two-stage process using an LM 3100 laboratory mill (Perten Instruments, Hägersten, Sweden) with a 0.7 mm sieve and a ZM 200 ultra-centrifugal mill (Retsch, Haan, Germany) with a 0.2 mm ring sieve to avoid excessive heat during milling. The flour was defatted twice with n-hexane at a ratio of 1:5 (*w*/*v*) by using a vortex mixer for 20 s, overhead-shaking (40 rpm, 60 min, 22 °C), and subsequent centrifugation (8000× *g*, 15 min, 4 °C). The fatty supernatant was discarded, and the pellet was dried overnight at 22 °C to allow excess n-hexane to evaporate. After complete drying, a mixture of CM (2:1) was added at a ratio of 1:10 (*w*/*v*). Preparation was performed twice by vortex mixing and overhead-shaking (40 rpm, 60 min, 22 °C), followed by centrifugation (8000× *g*, 15 min, 4 °C). The supernatants were pooled and transferred to a glass dish to increase the surface area for overnight CM evaporation at 22 °C. The solid residue was resuspended in 0.2 M phosphate-buffered saline (PBS, pH 7.5). This was achieved using an ultrasonic bath at a maximum temperature of 40 °C for 5 min, vortex mixing for 2 min, and overhead-shaking at 40 rpm for 30 min. After centrifugation (8000× *g*, 15 min, 4 °C), the supernatants containing nATIs were collected. To exclude impurities, further purification was performed by size-exclusion chromatography (SEC) using a 1260 Infinity LC system (Agilent, Santa Clara, CA, USA) equipped with a ShodexTM PROTEIN KW-802.5 gel filtration column (Resonac America, New York, NY, USA) under the following conditions: 25 °C column temperature, 1 mL min^−1^ flow rate, isocratic conditions (0.1 M PBS buffer, pH 7.5), and UV detection at 214 nm. The eluted fractions were collected and used for further analysis by electrophoresis (SDS-PAGE) and MALDI-TOF MS to identify high-purity nATI fractions.

**Figure 5:**
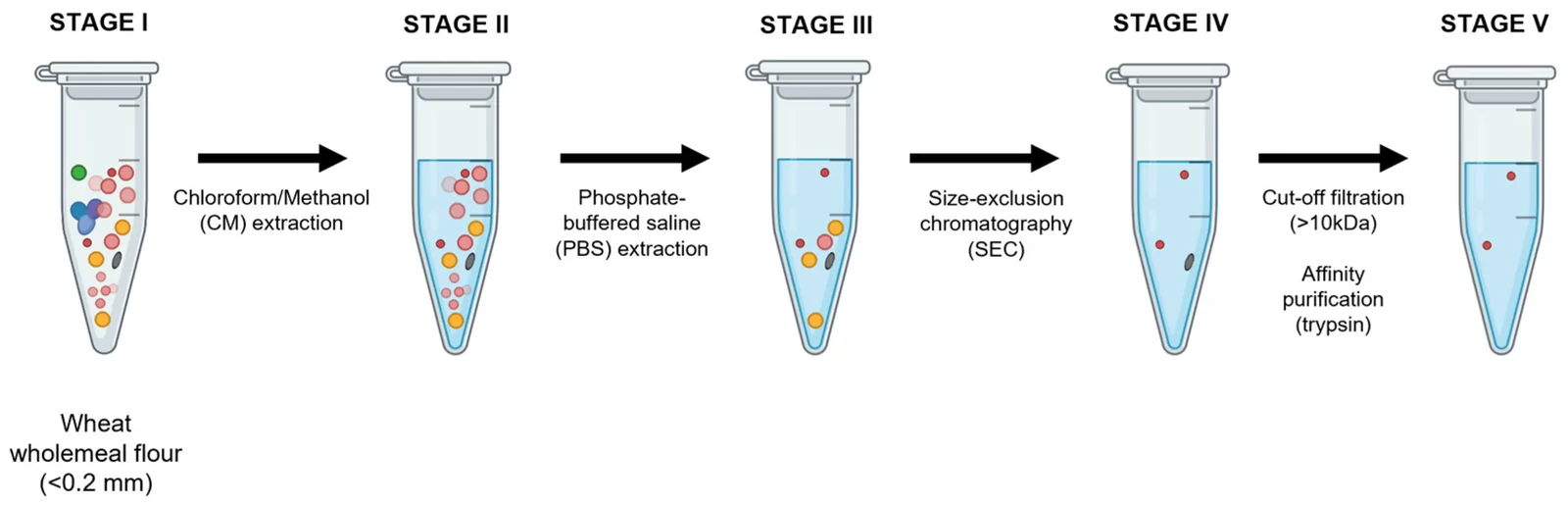
Schematic illustration of the preparation and purification procedure of nATIs from whole-grain wheat flour.

After chromatographic separation and analysis, the collected fractions were pooled according to their electrophoresis and mass pattern and concentrated using centrifugal filters with a cut-off of 10 kDa (Millipore, Merck, Burlington, VT, USA). Finally, affinity purification of nATIs was performed using TPCK-trypsin immobilized on magnetic beads (Takara Bio Europe, Saint-Germain-en-Laye, France). The clean-up procedure was performed according to the manufacturer’s instructions. After washing the magnetic beads, they were incubated with the nATI preparation at 37 °C for 20 min. Eventually, the magnetic beads were separated by centrifugation (10,000× *g*, 3 min) and washed repeatedly with ultrapure water (UPW) before desorbing the trypsin-bound proteins by lowering the pH with 0.1% trifluoroacetic acid (TFA) in PBS. The TFA was removed by evaporation to increase the stability of the proteins in solution. The nATI preparation was stored at −18 °C until further analysis. The workflow of the entire purification process is shown in Figure 5.

### 4.2. Heterologous Expression of Recombinant ATIs

#### 4.2.1. Plasmid Construction and Clone Selection

The coding sequences of the four target proteins 0.28 (P01083), 0.19 (P01085), CM1 (P16850), and CM3 (P17314) were obtained from the National Center for Biotechnology Information website (www.ncbi.nlm.nih.gov; accessed on 1 October 2021) and start for the mature forms of ATIs 0.28, CM1 and CM3 proteins at amino acid positions 31, 26, and 26, respectively, due to cleavage of the N-terminal signal peptide (Figure 3A). For expression in *E. coli*, the sequences for rATIs 0.28 and CM3 were cloned into the vector pET-DUET1. Polyhistidine tags (His6) were encoded in the 5′-region of the multiple cloning site I (MCS I) for subsequent affinity purification. Both ATI coding sequences were cloned in MCS I between *Sal*I/*Hin*dIII, and the designed plasmids were then produced by Biocat (Heidelberg, Germany). While CM3 was expressed in *E. coli* SHuffle T7 Competent (New England BioLabs, Ipswich, MA, USA), 0.28 was expressed in *E. coli* T7 Express Competent (New England Biolabs), which contains the chaperone-encoding plasmid pGRO7. The selection of these two *E. coli* strains was based on a preliminary screening comparing the expression yield of different strains and conditions.

For the expression of rATIs 0.19, CM1 and CM3 in *P. pastoris*, the expression vectors pPICZαA (for CM3 with His6 tag) and pPICZαC (Invitrogen, Groningen, The Netherlands) were used. The selected sequences were expressed both as fusion proteins with a C-terminal His6 tag (W His tag) and as proteins without an affinity tag (W/O His tag). The six cDNA constructs corresponding to the three rATI proteins (W and W/O His_6_ tag) were acquired from GENEWIZ (Leipzig, DE) and fused in-frame into the nucleotide sequence of the *Saccharomyces cerevisiae* α-factor secretion signal under the control of the AOX1 promoter of the pPICZαC expression vector. The resulting recombinant expression plasmids were linearized with *Pme*I and integrated into the *P. pastoris* genome by electroporation using a Multiporator^®^ (Eppendorf, Hamburg, Germany) at 1500 V for 5 ms. The *P. pastoris* strain GlcyoSwitch^®^ SuperMan5 (BioGrammatics, Carlsbad, CA, USA) was selected as the expression system as it was engineered to limit N-glycosylation to a maximum of five mannose residues. The sequences used for cloning and the plasmid maps are shown in Figure 3A, Figures S4 and S5.

#### 4.2.2. Large-Scale Expression of Recombinant ATI Proteins (rATIs)

For expression in *E. coli*, both strains (SHuffle T7 Competent and T7 Express Competent) were inoculated in LB (lysogeny broth) media containing the respective antibiotic (100 ppm ampicillin and 25 ppm chloramphenicol for 0.28, 100 ppm ampicillin for CM3) and incubated overnight at 37 °C. The next day, OD_600_ was determined and cultures were diluted in TB (terrific broth) media to a final OD_600_ of 0.12. Cultures were incubated at 37 °C and 160 rpm until the OD_600_ reached 0.8. Expression was induced by the addition of 1 mM IPTG. To induce the expression of the chaperone, 0.5 mM arabinose was also added to the culture expressing 0.28. The cultures were incubated at 30 °C for 4 h. Cells were then harvested by centrifugation at 3320× *g* for 30 min before resuspension in 0.2 M PBS (pH 7.5) and sonication (Branson Digital Sonifier W-250 D, Emerson Electric, St. Louis, MO, USA) at 50% amplitude for 10× 10 s with a 1 min pause between the pulses. The sonicated samples were centrifuged at 20,000× *g* for 15 min and the supernatant containing soluble proteins was transferred to another tube. The pellets were dissolved in urea buffer (8 M urea, 500 mM NaCl, 50 mM Na_2_HPO_4_) to resolve and examine insoluble proteins. The expression procedure was monitored by SDS-PAGE.

For expression in *P. pastoris*, the best transformants were selected based on a preliminary screening (2-step selection process). The first screening was performed using YPDS (Yeast Peptone Dextrose Sorbitol) plates containing 0.1 mg mL^−1^ and 0.5 mg mL^−1^ Zeocin^®^ (InvivoGen Europe, Toulouse, France), respectively, to select multicopy integrations. Then, MM (Minimal Methanol) plates were used to identify Mut^S^ (methanol utilization slow phenotype) transformants. For large-scale expression, aliquots of 4× 5 mL of BMGY (buffered glycerol-complex medium) were inoculated with the three selected highest-producing clones and incubated at 30 °C and 200 rpm for 7 h to prepare pre-cultures. The pre-cultures (20 mL each) were used for overnight cultures by inoculating 2 L of BMGY in 5 L baffled flasks and incubating at 30 °C and 200 rpm. When OD_600_ reached a value of 3, cultures were centrifuged (3320× *g*, 15 min, RT) and resuspended in 400 mL of BMMY (buffered methanol-complex medium) in 2 L baffled flasks. The cultures were incubated at 25 °C and 180 rpm for 3 days. The methanol content (1.5% *v*/*v*) was readjusted at specific time intervals and 500 µL aliquots were collected daily, centrifuged (14,000× *g*, 3 min) and used for SDS-PAGE to monitor the expression. Culture supernatants were obtained by centrifugation (6000× *g*, 15 min, RT) after three days of methanol induction. Before protein purification, supernatants were filtered using Nalgene^®^ sterile bottle top filters with a pore size of 0.2 µm (Carl Roth, Karlsruhe, Germany).

#### 4.2.3. Purification of Recombinant ATI Proteins

His6-tagged recombinant proteins were purified by FPLC^®^ using HisTrapTM FF crude columns (Cytiva—Global Life Sciences Solutions Austria, Pasching, Austria). To prepare His6-tagged proteins for FPLC purification, samples were adjusted to a pH of 7.8 with NaOH, centrifuged (6000× *g*, 15 min, RT), and filtered (0.2 µm). Supernatants were then purified in a single clean-up step with 1 mL or 5 mL HisTrapTM columns connected to an FPLC^®^ instrument (ÄKTA pure 25 M, GE Healthcare, Piscataway, NJ, USA) and equilibrated with a 0.05 M PBS (pH 7.8) containing 0.15 M NaCl and 0.005 M imidazole, while elution was performed using a 0.05 M PBS (pH 7.8) containing 0.15 M NaCl and 0.5 M imidazole at a flow rate of 1.5 mL min^−1^ (1 mL HisTrapTM column) and 5 mL min^−1^ (5 mL HisTrapTM column). Fractions of 1.5–1.8 mL were collected using a fraction collector connected to the FPLC^®^ instrument, pooled after SDS-PAGE analysis, and concentrated using Vivaspin^®^ centrifugal concentrators with a cut-off of 3 kDa (Sartorius, Goettingen, Germany).

Culture supernatants containing rATI proteins without His6 tags were prepared for ion-exchange chromatography (IEX) by concentrating them using Vivaspin^®^ centrifugal concentrators (3 kDa cut-off, Sartorius) to reduce the final volume. Subsequently, the concentrates were re-buffered in 50 mM Tris base (pH 8.4) using PD10 columns (Cytiva) and applied to a diethylaminoethyl cellulose (DEAE-C) column connected to the FPLC^®^ instrument. Equilibration was performed with a linear gradient using 50 mM Tris base (pH 8.4) mixed with 1 M NaCl at a flow rate of 1.5 mL min^−1^. Peak fractions were collected in 1.8 mL aliquots and analyzed by SDS-PAGE. Fractions containing the target IEX-purified proteins were subjected to a further purification step by size-exclusion chromatography (SEC), using a HiPrep 26/60 Sephacryl S-200 HR column (Cytiva) and 50 mM Tris buffer (pH 7.2) mixed with 0.15 M NaCl for elution at a flow rate of 1 mL min^−1^.

### 4.3. Protein Identification and Biochemical Characterization

#### 4.3.1. Electrophoresis and Mass Spectrometry

SDS-PAGE was performed to obtain general information on the expression yields, overall composition, and mass distribution of the crude and purified nATI and rATI preparations. SDS-PAGE analysis was performed in either 4–12%, 12% BoltTM (Invitrogen, Thermo Fisher Scientific, Waltham, MA, USA) or 15% handcast polyacrylamide gels using Bio-Rad Laboratories (Munich, Germany) equipment. Briefly, protein samples were mixed with 4× Laemmli buffer and reducing agent according to the manufacturer’s instructions before heating at 98 °C for 3 min and centrifugation (tabletop centrifuge). Samples (10–40 µL) and molecular weight markers were loaded, and the gel was run at 165 V for 35–120 min. For protein visualization, proteins separated by electrophoresis were stained with a Coomassie blue solution. Gels were destained by successive washing with water.

The exact masses of the nATI preparation and rATI proteins were further determined using a MALDI-TOF mass spectrometer (Bruker LT Microflex, Billerica, MA, USA). Protein suspensions were diluted 1:1 with acetonitrile (ACN) containing 0.1% TFA, applied to the MALDI steel plate, and evaporated at RT before being covered with a matrix solution consisting of 10 mg sinapic acid in 1 mL H_2_O/ACN (50:50) and 0.1% TFA. The laser power was set to 100%, and the number of shots was varied to obtain clear spectra with a satisfactory signal-to-noise ratio.

To determine the composition of the nATI preparation and to confirm the identity of the rATIs, purified preparations were analyzed by LC-MS/MS using a Waters H-class UPLC system equipped with a Xevo G2 QTOF mass spectrometer (Waters Corporation, Milford, CT, USA). Before LC-MS/MS detection, protein preparations were mixed with 0.1% RapiGest SF Surfactant (Waters, Vienna, Austria) and proteins were reduced by the addition of 50 mM dithioerythritol (DTE) at 60 °C for 20 min. Alkylation of the proteins was performed with 100 mM iodoacetamide for 30 min in the dark. For protein digestion, 20 µL of trypsin protease (MS grade, Thermo Fisher Scientific) was added before incubation at 37 °C and 300 rpm for 16 h. The tryptic digestion was stopped by adding 10 µL of 5% TFA, and the resulting peptides were purified with stage-tips containing 5 layers of CDS Empore^TM^ SDB-XC extraction disks (3M, Saint Paul, MN, USA). The stage tips were equilibrated with methanol before use, loaded with sample peptides, and washed twice with H_2_O/ACN/TFA (97.5:2.5:0.1) before the peptides were eluted with H_2_O/ACN/TFA (40:60:0.1). The eluate was evaporated at 45 °C by vacuum centrifugation, resuspended in H_2_O/ACN/TFA (90:10:0.1), and injected into the UPLC system using a CSH C18 column (150 × 1.0 mm, 1.7 µm, Waters). Data acquisition was performed from 350 to 1850 *m*/*z*, and up to 12 MS/MS ions were fragmented by mass-dependent high-energy ramping. The recorded spectra were processed with ProteinLynx Global SERVER^TM^ software version 3.0.3 (PLGS, supplied by Waters Corporation), and the data were used for identification via MASCOT MS/MS ion search.

#### 4.3.2. Immunoassays

For the detection of wheat allergens other than ATIs in the nATI preparation, an enzyme-linked immunosorbent assay (ELISA) was performed. ELISA plates (96-well plates) were coated with purified nATI preparation (conc.: 5 µg mL^−1^, 100 µL per well, incubated overnight at 4 °C) before washing with PBST (phosphate-buffered saline with 0.05% Tween 20, 2×) and blocking with 1% BSA in PBST (200 µL per well, incubation at 37 °C for 2 h). Eventually, different rabbit sera were added containing polyclonal antibodies (pAb) specific for different wheat allergens, including profilin (Tri a 12), non-specific lipid transfer protein (nsLTP, Tri a 14), beta-amylase (Tri a 17), gamma-gliadin (Tri a 20), thioredoxin (Tri a 25), high-molecular-weight glutenin (HMW, Tri a 26), 1-cys-peroxiredoxin (Tri a 32), dehydrin (Tri a 35), low-molecular-weight glutenin (LMW, Tri a 36), alpha-purothionin (Tri a 37), serine protease inhibitor-like protein (Tri a 39), or avenin-like protein (ALP) (1:10,000 in 0.5% BSA in PBST, 100 µL per well, followed by incubation overnight at 4 °C). Plates were washed with PBST (5×) before adding HRP (horse radish peroxidase)-labeled goat anti-rabbit IgG antibodies (Thermo Fisher Scientific, 1:2000 in 0.5% BSA in PBST, 100 µL per well, incubation at 37 °C for 30 min and at 4 °C for 30 min). After final washing with PBST (5×), ABTS/citric acid/H_2_O_2_ substrate solution (Thermo Fisher Scientific) was added, and plates were incubated in the dark for 10 min to allow the development of the green end product. Finally, absorption was measured in a range between 405 nm and 490 nm. The procedure was performed simultaneously with positive (recombinant allergens corresponding to respective pAbs, wheat preparation (PBS)) and negative (buffer) controls.

#### 4.3.3. Circular Dichroism Analysis

CDA was used for estimating the overall secondary structure composition of the recombinant proteins to verify their correct folding during heterologous expression. For CDA, proteins were re-buffered in 10 mM Na-phosphate buffer in the absence of NaCl as chloride ions show high absorption in the far-UV region [45]. Quartz glass cuvettes with a path length of 1 mm were used, and spectra were recorded in the range from 190 nm to 260 nm using a J-810 spectropolarimeter (JASCO, Tokyo, Japan). Spectra were recorded at a scanning speed of 50 nm min^−1^ at room temperature with a resolution of 0.5 nm and corrected by subtracting the corresponding buffer spectrum obtained under identical conditions. The results are averages of three measurements and are expressed as mean residue ellipticity [ø]. The online server DICHROWEB and its embedded algorithms were used to obtain an estimate of the secondary structure composition of the proteins.

#### 4.3.4. Functionality Assays

The functionality of rATIs and nATIs was tested by evaluating their inhibitory potential against commercial α-amylase and trypsin. The α-amylase inhibitory activity (AIA) was determined using a DNS assay with maltose as substrate [46], and the trypsin inhibitory activity (TIA) was determined by an enzymatic assay with the chromogenic substrate N_α_-benzoyl-L-arginine-4-nitroanilide-hydrochloride (L-BAPA) [47].

### 4.4. Microarray-Based Allergy Diagnosis

A wheat allergen microarray assay was designed and printed on a SciFlexArrayer S12 (Scienion, Berlin, Germany) following a previously developed protocol [48]. Briefly, MCP-2 (Lucidant Polymers, Sunnyvale, CA, USA)-coated glass slides (Marienfeld, Lauda-Königshofen, Germany) fitting six microarrays were spotted with the recombinant and natural ATIs, plus a range of other recombinant wheat allergens and synthetic wheat allergen-derived peptides [49], at a concentration of 1 mg mL^−1^ in 75 mM phosphate buffer, pH 8.4, in triplicates. The reactivity of serum IgE, IgG, IgG1, IgG4, and IgA to the microarrayed allergens and peptides was determined as follows: the glass slides were washed in PBST (0.5% Tween 20), dried by centrifugation and, subsequently, 30 µL of the serum sample was added. Sera were undiluted for IgE detection, and diluted 1:50 for IgG and IgG1, 1:20 for IgG4 and 1:10 for IgA detection in sample diluent (Thermo Fisher Scientific, Uppsala, Sweden). After washing, secondary antibodies were applied, diluted to a concentration of 1.8 µg mL^−1^ in sample diluent (anti-human IgE: Roche, Mannheim, Germany; anti-human IgG: Roche; anti-human IgG1: Invitrogen, Waltham, MA, USA; anti-human IgG4: BD Biosciences, San Diego, CA, USA; anti-human IgA1/A2: BD Biosciences). All secondary antibodies were labeled with Dylight 550 (Pierce, Rockford, IL, USA) according to the manufacturer’s protocol. Slides were washed with PBST, dried by centrifugation, and scanned using a confocal laser scanner (Tecan, Männedorf, Switzerland). MAPIX analysis software version 8.5.0 (Innopsys, Carbonne, France) was used for image analysis. Conversion of fluorescence units to ISAC standard units (ISU) was performed as previously described [50], using the above-mentioned allergens and corresponding human sera for calibration of IgE, IgG, IgG4, and IgA measurements. For the calibration of IgG1 measurements, human TNFα protein (Active) ab259410 (Abcam, Amsterdam, The Netherlands) was spotted onto the microarray in three dilutions, 1:250,000, 1:600,000 and 1:1.2 Mio and detected with an anti-TNFα IgG1 antibody of known concentration (Merck Serono, Corsier-sur-Vevey, Switzerland).

Serum samples from 94 wheat-sensitized Japanese children were provided by NHO Sagamihara National Hospital, Japan. Sensitization to wheat, as determined by ImmunoCAP™ (f4) (Thermo Fisher Scientific), was defined as wheat-specific IgE level > 0.35 kU_A_ L^−1^. Blood samples were collected 1–3 months before an oral food challenge (OFC) with increasing amounts of udon noodles (wheat flour, salt, water) up to a maximal dose of 100 g. The OFC was conducted in order to categorize the immune responses of the patients.

The IgE-binding activity of 80 of the 94 serum samples was additionally tested by the ImmunoCAP™ ISAC E112i assay (Thermo Fisher Scientific), which contains, among 112 allergens, a natural ATI protein from wheat (nTri a aA_TI).

All statistical analyses were carried out in R version 4.5.0 (R Core Team, Vienna, Austria) using the packages ggpubr (version 1.0.0), ggplot2 (version 4.0.3) and ggsignif (version 0.6.4). The significance of differences between the two OFC response groups was tested by Wilcoxon signed-rank test (Mann–Whitney U-test).

## 5. Conclusions

This study underscores the challenge of achieving a pure ATI preparation for WRD testing. The results shown in this study prove that a simple ATI purification does not ensure the complete elimination of other wheat proteins that could potentially affect the immunological properties of the preparation. An elaborate ATI isolation and purification process resulting in satisfying purity, however, was shown to be both time-consuming and material-intensive, with a relatively modest yield of ATIs. Given these facts, the synthesis of recombinant ATI proteins in heterologous expression systems should be considered as an alternative approach, offering a promising avenue for future research. Due to the complex structure of ATIs stabilized by disulfide bonds, their expression in eukaryotic systems (e.g., yeasts such as *P. pastoris*) is favored over prokaryotic systems (e.g., *E. coli*), as demonstrated in this study. Although expression in *P. pastoris* yielded high levels of soluble ATIs 0.19, CM1, and CM3, the results of the CD analysis and inhibitory assays indicate that the recombinant proteins were not properly folded (as predicted for native wheat ATIs). However, immunoreactive evaluation using microarrays showed that the recombinant ATI proteins are capable of inducing allergen-specific antibody responses in a considerable subset of wheat-allergic individuals. Although the recombinant proteins produced in this study may not completely reflect native ATIs originating from wheat, they can be used to produce monoclonal or polyclonal antibodies that can be applied in immunoassays to qualitatively and quantitatively assess ATIs in different wheat genotypes or wheat-derived foods, or to isolate native ATIs from wheat by affinity purification.

## Figures and Tables

**Table 1:** Identification of proteins present in the final nATI preparation by LC-MS/MS analysis and comparison with the SwissProt database (MASCOT MS/MS ion search).

Identified Protein	Entry Name	Score	Peptides
Alpha-amylase inhibitor 0.19 OS = *Triticum aestivum* OX = 4565 PE = 1 SV = 1 *	IAA1_WHEAT	1505	5
Alpha-amylase inhibitor 0.53 OS = *Triticum aestivum* OX = 4565 PE = 1 SV = 1 *	IAA5_WHEAT	1201	4
Alpha-amylase/trypsin inhibitor CM3 OS = *Triticum aestivum* OX = 4565 PE = 1 SV = 1 *	IAAC3_WHEAT	541	4
Alpha-amylase inhibitor 0.28 OS = *Triticum aestivum* OX = 4565 GN = IMA1 PE = 1 SV = 3 *	IAA2_WHEAT	524	3
Alpha-amylase/trypsin inhibitor CM16 OS = *Triticum aestivum* OX = 4565 PE = 1 SV = 1 *	IAC16_WHEAT	351	3
Alpha-amylase inhibitor WDAI-3 (Fragment) OS = *Triticum aestivum* OX = 4565 GN = IHA-B1–2 PE = 1 SV = 1	IAA3_WHEAT	328	1
Non-specific lipid-transfer protein 2G OS = Triticum aestivum OX = 4565 PE = 1 SV = 2	NLT2G_WHEAT	123	1
Chymotrypsin inhibitor WCI OS = *Triticum aestivum* OX = 4565 PE = 1 SV = 1	ICIW2_WHEAT	54	1
Alpha-amylase/trypsin inhibitor CM2 OS = *Triticum aestivum* OX = 4565 PE = 1 SV = 2	IAAC2_WHEAT	40	2
Thioredoxin H-type OS = *Triticum aestivum* OX = 4565 PE = 2 SV = 3	TRXH_WHEAT	30	1
Avenin-like b10 OS = *Triticum aestivum* OX = 4565 PE = 3 SV = 1	AVLBA_WHEAT	23	1

* Protein sequences with at least two identified peptides and scores > 60 are classified as clearly identified.

## Data Availability

The original data presented in the study are openly available in the Zenodo repository: (i) Raw data files of the LC-MS/MS of the natural wheat ATI preparation at https://doi.org/10.5281/zenodo.17643329; (ii) MALDI-TOF data at https://doi.org/10.5281/zenodo.17643584; (iii) data on the oral food challenge, allergy response and immunoreactivity microarray assay of Japanese children and original gels and blots of figures included in this article at https://doi.org/10.5281/zenodo.17482282.

## Supplementary Materials

The following supporting information can be downloaded at: https://www.mdpi.com/article/10.3390/ijms27198857/s1.

## Abbreviations

The following abbreviations are used in this manuscript:

ALP	Avenin-like proteins
ATI	Amylase–trypsin inhibitor
CD	Celiac disease
CDA	Circular dichroism analysis
DNS	Dinitrosalicylic acid
IBS	Irritable bowel syndrome
IEX	Ion exchange chromatography
NCWS	Non-celiac wheat sensitivity
nsLTP	Non-specific lipid transfer proteins
SEC	Size exclusion chromatography
TIA	Trypsin inhibitory activity
WA	Wheat allergy
WDEIA	Wheat-dependent exercise-induced anaphylaxis
WRD	Wheat-related disorder

## References

[1] Fjeldheim Dale H., Biesiekierski J.R., Lied G.A. (2019). Non-coeliac gluten sensitivity and the spectrum of gluten-related disorders: An updated overview. Nutr. Res. Rev..

[2] Sapone A., Ba J.C., Ciacci C., Dolinsek J., Green P.H.R., Hadjivassiliou M., Kaukinen K., Rostami K., Sanders D.S., Schumann M. (2012). Spectrum of gluten-related disorders: Consensus on new nomenclature and classification. BMC Med..

[3] Aziz I. (2018). The global phenomenon of self-reported wheat sensitivity. Am. J. Gastroenterol..

[4] Neyer A., Dölle-Bierke S., Höfer V., Grünhagen J., Beyer K., Worm M. (2025). Prevalence and clinical symptoms of wheat allergy in adults and adolescents in Central Europe. Clin. Exp. Allergy.

[5] Størdal K., Kurppa K. (2025). Celiac disease, non-celiac wheat sensitivity, wheat allergy—Clinical and diagnostic aspects. Semin. Immunol..

[6] Sicherer S.H., Sampson H.A. (2014). Food allergy: Epidemiology, pathogenesis, diagnosis, and treatment. J. Allergy Clin. Immunol..

[7] Catassi C., Alaedini A., Bojarski C., Bonaz B., Bouma G., Carroccio A., Castillejo G., De Magistris L., Dieterich W., Di Liberto D. (2017). The overlapping area of non-celiac gluten sensitivity (NCGS) and wheat-sensitive irritable bowel syndrome (IBS): An update. Nutrients.

[8] Lebwohl B., Sanders D.S., Green P.H.R. (2018). Coeliac disease. Lancet.

[9] Zhu Y.Q., Wang D.Q., Liu B., Hu Y., Shen Y.Y., Xu J.H., Tang H. (2020). Wheat-dependent exercise-induced anaphylaxis in Chinese people: A clinical research on 33 cases with antigenic analysis of wheat proteins. Clin. Exp. Dermatol..

[10] An C., Yang J., Pinto-Sanchez M.I., Verdu E.F., Lebwohl B., Green P.H., Alaedini A. (2025). Molecular triggers of non-celiac wheat sensitivity: A scoping review and analysis. Am. J. Gastroenterol..

[11] van Gils T., Simrén M. (2025). The role of gluten and wheat in irritable bowel syndrome and noncoeliac gluten or wheat sensitivity. Curr. Opin. Gastroenterol..

[12] Scherf K.A. (2019). Immunoreactive cereal proteins in wheat allergy, non-celiac gluten/wheat sensitivity (NCGS) and celiac disease. Curr. Opin. Food Sci..

[13] Ryan C.A. (1990). Protease inhibitors in plants: Genes for improving defenses against insects and pathogens. Annu. Rev. Phytpathol..

[14] Carbonero P., Díaz I., Vicente-Carbajosa J., Alfonso-Rubí J., Gaddour K., Lara P., Scarascia Mugnozza G.T., Porceddu E., Pagnotta M.A. (1999). Cereal α-amylase/trypsin inhibitors and transgenic insect resistance. Genetics and Breeding for Crop Quality and Resistance.

[15] Geisslitz S., Shewry P., Brouns F., America A.H.P., Caio G.P.I., Daly M., D’Amico S., De Giorgio R., Gilissen L., Grausgruber H. (2021). Wheat ATIs: Characteristics and role in human disease. Front. Nutr..

[16] Sanchez-Monge R., Gomez L., Barber D., Lopez-Otin C., Armentia A., Salcedo G. (1992). Wheat and barley allergens associated with baker’s asthma. Glycosylated subunits of the α-amylase-inhibitor family have enhanced IgE-binding capacity. Biochem. J..

[17] Junker Y., Zeissig S., Kim S.J., Barisani D., Wieser H., Leffler D.A., Zevallos V., Libermann T.A., Dillon S., Freitag T.L. (2012). Wheat amylase trypsin inhibitors drive intestinal inflammation via activation of toll-like receptor 4. J. Exp. Med..

[18] Bellinghausen I., Weigmann B., Zevallos V., Maxeiner J., Reißig S., Waisman A., Schuppan D., Saloga J. (2019). Wheat amylase-trypsin inhibitors exacerbate intestinal and airway allergic immune responses in humanized mice. J. Allergy Clin. Immunol..

[19] Huebener S., Tanaka C.K., Uhde M., Zone J.J., Vensel W.H., Kasarda D.D., Beams L., Briani C., Green P.H.R., Altenbach S.B. (2015). Specific nongluten proteins of wheat are novel target antigens in celiac disease humoral response. J. Proteome Res..

[20] Sánchez D., Štěpánová Honzová S., Hospodková M., Hoffmanová I., Hábová V., Halada P., Tlaskalová-Hogenová H., Tučková L. (2018). Occurrence of serum antibodies against wheat alpha-amylase inhibitor 0.19 in celiac disease. Physiol. Res..

[21] Breiteneder H., Radauer C. (2004). A classification of plant food allergens. J. Allergy Clin. Immunol..

[22] Tundo S., Lupi R., Lafond M., Giardina T., Larré C., Denery-Papini S., Morisset M., Kalunke R., Sestili F., Masci S. (2018). Wheat ATI CM3, CM16 and 0.28 allergens produced in *Pichia pastoris* displays a different eliciting potential in food allergy to wheat. Plants.

[23] Tokmakov A.A., Kurotani A., Takagi T., Toyama M., Shirouzu M., Fukami Y., Yokoyama S. (2012). Multiple post-translational modifications affect heterologous protein synthesis. J. Biol. Chem..

[24] Altmann F. (2007). The role of protein glycosylation in allergy. Int. Arch. Allergy Immunol..

[25] Fu L., Wang R., Zhou J., Wang C., Wang Y. (2023). Site-specific N-glycosylation characterization and allergenicity analysis of globulin-1 S allele from wheat. Food Sci. Hum. Wellness.

[26] Altenbach S.B., Vensel W.H., Dupont F.M. (2011). The spectrum of low molecular weight α-amylase/protease inhibitor genes expressed in the US bread wheat cultivar Butte 86. BMC Res. Notes.

[27] Geisslitz S., Longin F.H., Koehler P., Scherf K.A. (2020). Comparative quantitative LC-MS/MS analysis of 13 amylase/trypsin inhibitors in ancient and modern *Triticum* species. Sci. Rep..

[28] Cuccioloni M., Mozzicafreddo M., Bonfili L., Cecarini V., Giangrossi M., Falconi M., Saitoh S.-I., Eleuteri A.M., Angeletti M. (2017). Interfering with the high-affinity interaction between wheat amylase trypsin inhibitor CM3 and toll-like receptor 4: In silico and biosensor-based studies. Sci. Rep..

[29] Ferranti P., Mamone G., Picariello G., Addeo F. (2007). Mass spectrometry analysis of gliadins in celiac disease. J. Mass Spectrom..

[30] Zevallos V.F., Raker V., Tenzer S., Jimenez-Calvente C., Ashfaq-Khan M., Rüssel N., Pickert G., Schild H., Steinbrink K., Schuppan D. (2017). Nutritional wheat amylase-trypsin inhibitors promote intestinal inflammation via activation of myeloid cells. Gastroenterology.

[31] Geisslitz S., Weegels P., Shewry P., Zevallos V., Masci S., Sorrells M., Gregorini A., Colomba M., Jonkers D., Huang X. (2022). Wheat amylase/trypsin inhibitors (ATIs): Occurrence; function and health aspects. Eur. J. Nutr..

[32] Krouch D., Vreeke G.J.C., America A.H.P., Mes J.J., Wierenga P.A., Vincken J.-P., Bastiaan-Net S., Weegels P.L. (2025). Amylase trypsin inhibitors activation of toll-like receptor 4 revisited: The dominance of lipopolysaccharides contamination. Int. J. Biol. Macromol..

[33] Weichel M., Vergoossen N.J., Bonomi S., Scibilia J., Ortolani C., Ballmer-Weber B.K., Pastorello E.A., Crameri R. (2006). Screening the allergenic repertoires of wheat and maize with sera from double-blind, placebo-controlled food challenge positive patients. Allergy.

[34] García-Maroto F., Carbonero P., García-Olmedo F. (1991). Site-directed mutagenesis and expression in *Escherichia coli* of WMAI-1; a wheat monomeric inhibitor of insect α-amylase. Plant Mol. Biol..

[35] Okuda M., Satoh T., Sakurai N., Shibuya K., Kaji H., Samejima T. (1997). Overexpression in *Escherichia coli* of chemically synthesized gene for active 0.19 α-amylase inhibitor from wheat kernel. J. Biochem..

[36] Woestenenk E.A., Hammarströn M., van den Berg S., Härd T., Berglund H. (2004). His tag effect on solubility of human proteins produced in *Escherichia coli*: A comparison between four expression vectors. J. Struct. Funct. Genom..

[37] Oda Y., Matsunaga T., Fukuyama K., Miyazaki T., Morimoto T. (1997). Tertiary and quaternary structures of 0.19 α-amylase inhibitor from wheat kernel determined by X-ray analysis at 2.06 Å resolution. Biochemistry.

[38] Lee J.R., Lee S.S., Jang H.H., Lee Y.M., Park J.H., Park S.-C., Moon J.C., Park S.K., Kim S.Y., Lee S.Y. (2009). Heat-shock dependent oligomeric status alters the function of a plant-specific thioredoxin-like protein; AtTDX. Proc. Natl. Acad. Sci. USA.

[39] Sanz-Barrio R., Fernández-San Millán A., Carballeda J., Corral-Martínez P., Seguí-Simarro J.M., Farran I. (2012). Chaperone-like properties of tobacco plastid thioredoxins f and m. J. Exp. Bot..

[40] Du H., Kim S., Hur Y.S., Lee M.S., Lee S.H., Cheon C.I. (2015). A cytosolic thioredoxing acts as a molecular chaperone for peroxisome matrix proteins as well as antioxidant in peroxisome. Mol. Cells.

[41] Valenta R., Hochwallner H., Linhart B., Pahr S. (2015). Food allergies: The basics. Gastroenterology.

[42] Thiel A.L., Ragab M., Wagner A.E., Divanovic S., Derer S., Sina C. (2020). Purification and functional characterization of the chloroform/methanol-soluble protein 3 (CM3) from *Triticum aestivum* in *Drosophila melanogaster*. Front. Nutr..

[43] Sun R., Qian M.G., Zhang X. (2024). T and B cell epitope analysis for the immunogenicity evaluation and mitigation of antibody-based therapeutics. mAbs.

[44] Du H., Mallik L., Hwang D., Sun Y., Kaku C., Hoces D., Sun S.M., Ghinnagow R., Carro S.D., Phan H.A.T. (2025). Targeting peptide antigens using a multiallelic MHC I-binding system. Nat. Biotechnol..

[45] Kelly S.M., Jess T.J., Price N.C. (2005). How to study proteins by circular dichroism. Biochim. Biophys. Acta Proteins Proteom..

[46] Gélinas P., Gagnon F. (2018). Inhibitory activity towards human α-amylase in wheat flour and gluten. Int. J. Food Sci. Technol..

[47] Call L., Reiter E.V., Wenger-Oehn G., Strnad I., Grausgruber H., Schoenlechner R., D’Amico S. (2019). Development of an enzymatic assay for the quantitative determination trypsin inhibitory activity in wheat. Food Chem..

[48] Gattinger P., Niespodziana K., Stiasny K., Sahanic S., Tulaeva I., Borochova K., Dorofeeva Y., Schlederer T., Sonnweber T., Hofer G. (2022). Neutralization of SARS-CoV-2 requires antibodies against conformational receptor-binding domain epitopes. Allergy.

[49] Krisai A., Call L.M., Huang H.J., Schlederer T., Tulaev M., Focke-Tejkl M., Linhart B., Valenta R., Grausgruber H. (2022). Development of a wheat allergen chip for analysis of wheat allergen-specific antibody responses. Eur. J. Immunol..

[50] Lupinek C., Wollmann E., Baar A., Banerjee S., Breiteneder H., Broecker B.M., Bublin M., Curin M., Flicker S., Garmatiuk T. (2024). Advances in allergen-microarray technology for diagnosis and monitoring of allergy: The MeDALL allergen-chip. Methods.

