# Supplementary material for: Heterologous Expression and Immunoreactivity of Wheat Amylase–Trypsin Inhibitors
Source: Int J Mol Sci. 2026 Oct 4;27(19):8857. doi: 10.3390/ijms27198857 (PMC13664919; doi:10.3390/ijms27198857)
Supplement: Supplementary file 1 [file ijms-27-08857-s001.zip › ijms-4514906-supplementary.pdf]

Heterologous expression and immunoreactivity of wheat amylase-trypsin inhibitors

Lisa Call[1*§], Thomas Svoboda[1§], Andrea Krisai[1], Tim Hacker[1], Kathrin Lauter[1], Stefano D'Amico[2], Birgit Linhart[3], Agnès Amouric[4†], Mickaël Lafond[4‡], Thierry Giardina[4], Motohiro Ebisawa[5], Rudolf Valenta[3,6], Heinrich Grausgruber[1]

[1] Department of Agricultural Sciences, BOKU University, 3430 Tulln an der Donau, Austria

[2] AGES - Austrian Agency for Health and Food Safety, 1220 Vienna, Austria

[3] Institute of Pathophysiology and Allergy Research, Medical University of Vienna, 1090 Vienna, Austria

[4] Institut des Sciences Moléculaires de Marseille (iSm2), Aix-Marseille Université, CNRS, Central Marseille, 13397 Marseille, France

[5] Clinical Research Center for Allergy and Rheumatology, NHO Sagamihara National Hospital, Sagamihara, Kanagawa 252-0392, Japan

[6] Center for Molecular Allergology, Karl Landsteiner University for Health Sciences, 3500 Krems an der Donau, Austria

Corresponding author: Heinrich Grausgruber (heinrich.grausgruber@boku.ac.at)

[*] current address: AGES - Austrian Agency for Health and Food Safety, Vienna, Austria

[†] current address: Aix-Marseille Université, CNRS, IMM-FR3479, Marseille, France

[‡] current address: INRAE, Aix-Marseille University, Biodiversité et Biotechnologie Fongique, Marseille, France

[§] these authors contributed equally to this work

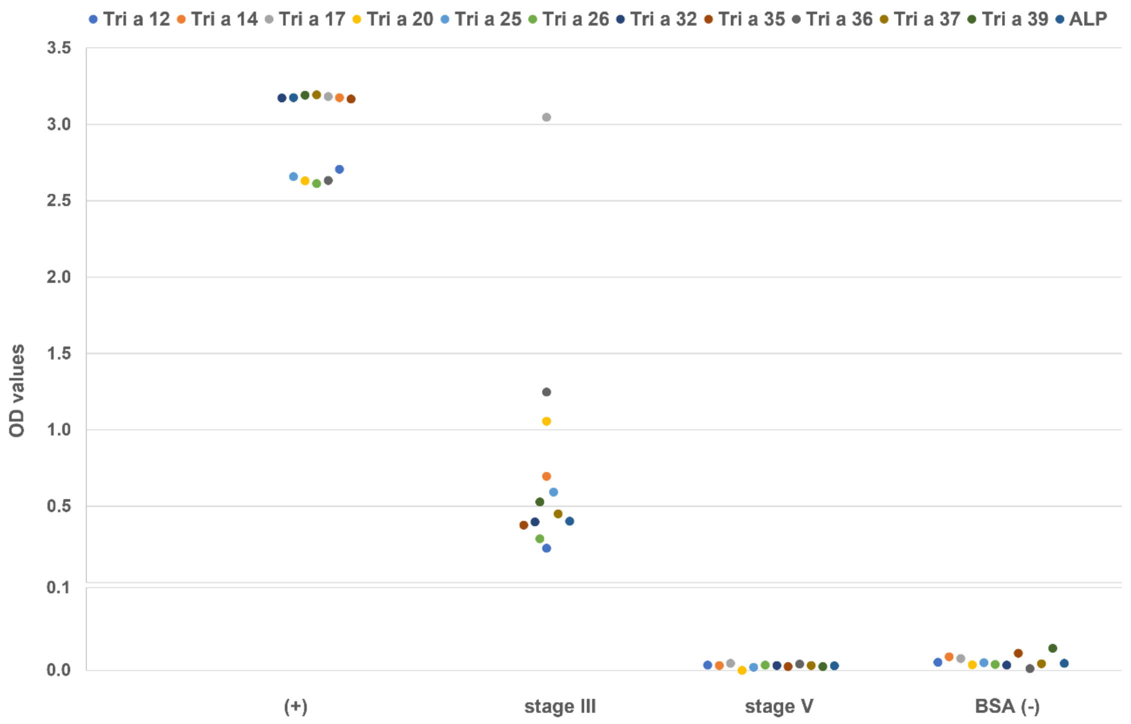

Supplementary Figure S1. Results obtained from ELISA analysis to test the purity of the nATI preparation. Reactivity (OD values, y-axis) of polyclonal rabbit antisera raised against the wheat allergens profilin (Tri a 12), non-specific lipid transfer protein (nsLTP, Tri a 14), beta-amylase (Tri a 17), gamma-gliadin (Tri a 20), thioredoxin (Tri a 25), high-molecular-weight glutenin (HMW, Tri a 26), 1-cys-peroxiredoxin (Tri a 32), dehydrin (Tri a 35), low-molecular-weight glutenin (LMW, Tri a 36), alpha-purothionin (Tri a 37), serine protease inhibitor-like protein (Tri a 39), or avenin-like protein (ALP) to the nATI preparations stages III and V, and the corresponding wheat allergens (+) as positive controls (x-axis) was measured by ELISA. The extract at stage V of the purification procedure represents the final nATI preparation. Bovine serum albumin (BSA) was additionally coated and tested as a negative control (-). Values from blank measurements (buffer) were subtracted from all sample values.

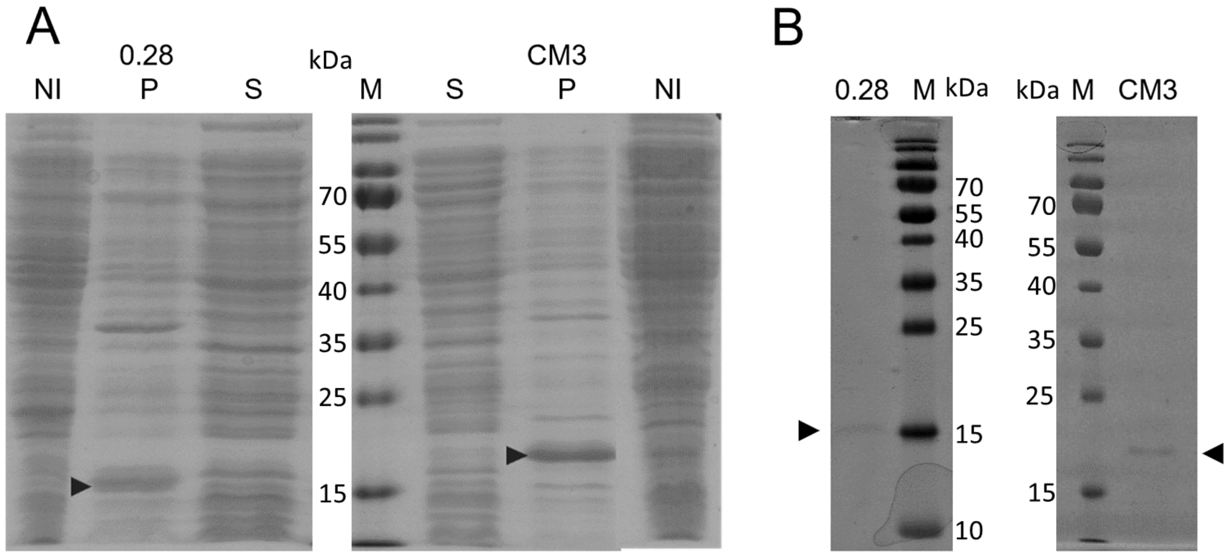

Supplementary Figure S2. SDS-PAGE analysis of the composition of recombinant ATIs expressed in *Escherichia coli*: (**A**) unpurified recombinant ATIs (rATIs) 0.28 and CM3 (S, soluble PBS fraction after sonication; P, insoluble fraction (pellet) resuspended in 8M urea; NI, non-induced control in which no IPTG was added); (**B**) rATIs 0.28 and CM3 after affinity purification. Black arrows indicate the expected size of ATI 0.28 (16.8 kDa) and CM3 (19.3 kDa). Lane M comprises marker polypeptides with the following molecular weights: 180, 130, 100, 70, 55, 40, 35, 25, 15, and 10 kDa (Thermo Scientific™ PageRuler™ Prestained Protein Ladder).

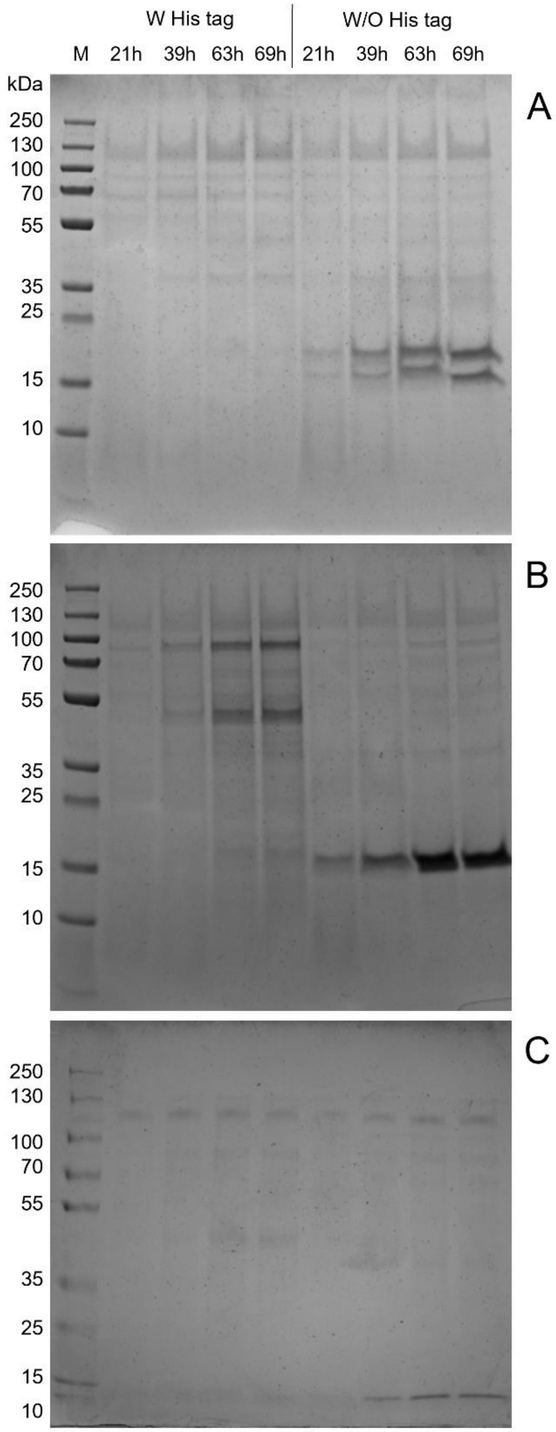

Supplementary Figure S3. SDS-PAGE monitoring of ATIs expressed in *Pichia pastoris* with (W) and without (W/O) His tags at different time intervals: (**A**) 0.19; (**B**) CM1; (**C**) CM3. M comprises marker polypeptides with the following molecular weights: 250, 130, 100, 70, 55, 40, 35, 25, 15, and 10 kDa (Thermo Scientific™ PageRuler™ Plus Prestained Protein Ladder).

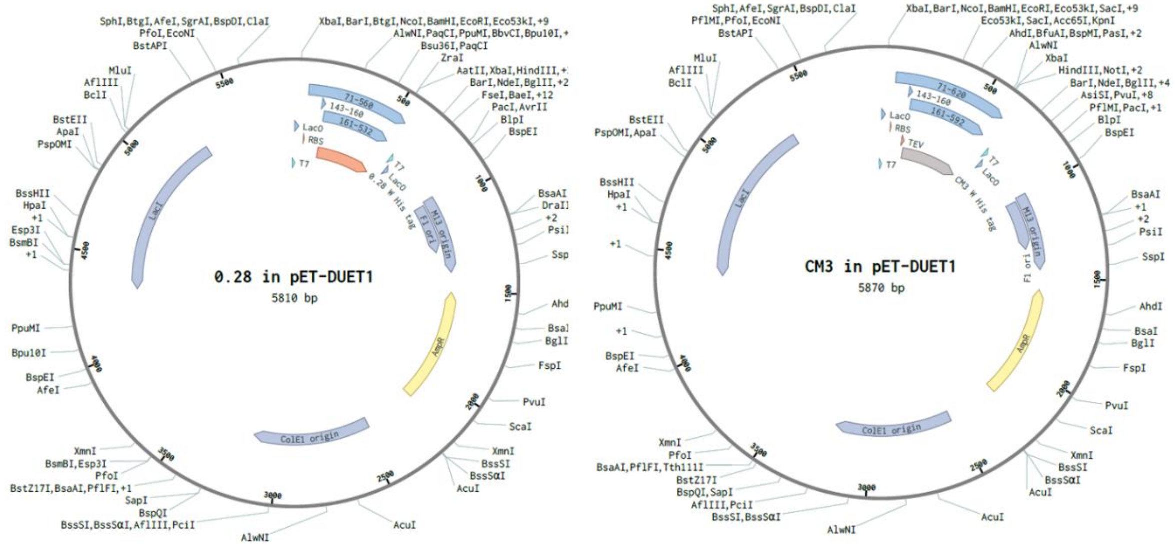

Supplementary Figure S4. Plasmid maps of the pET-DUET1 expression vector including amino acid sequences of ATIs 0.28 and ATI CM3 (with His_6 tag) for heterologous expression in *Escherichia coli*.

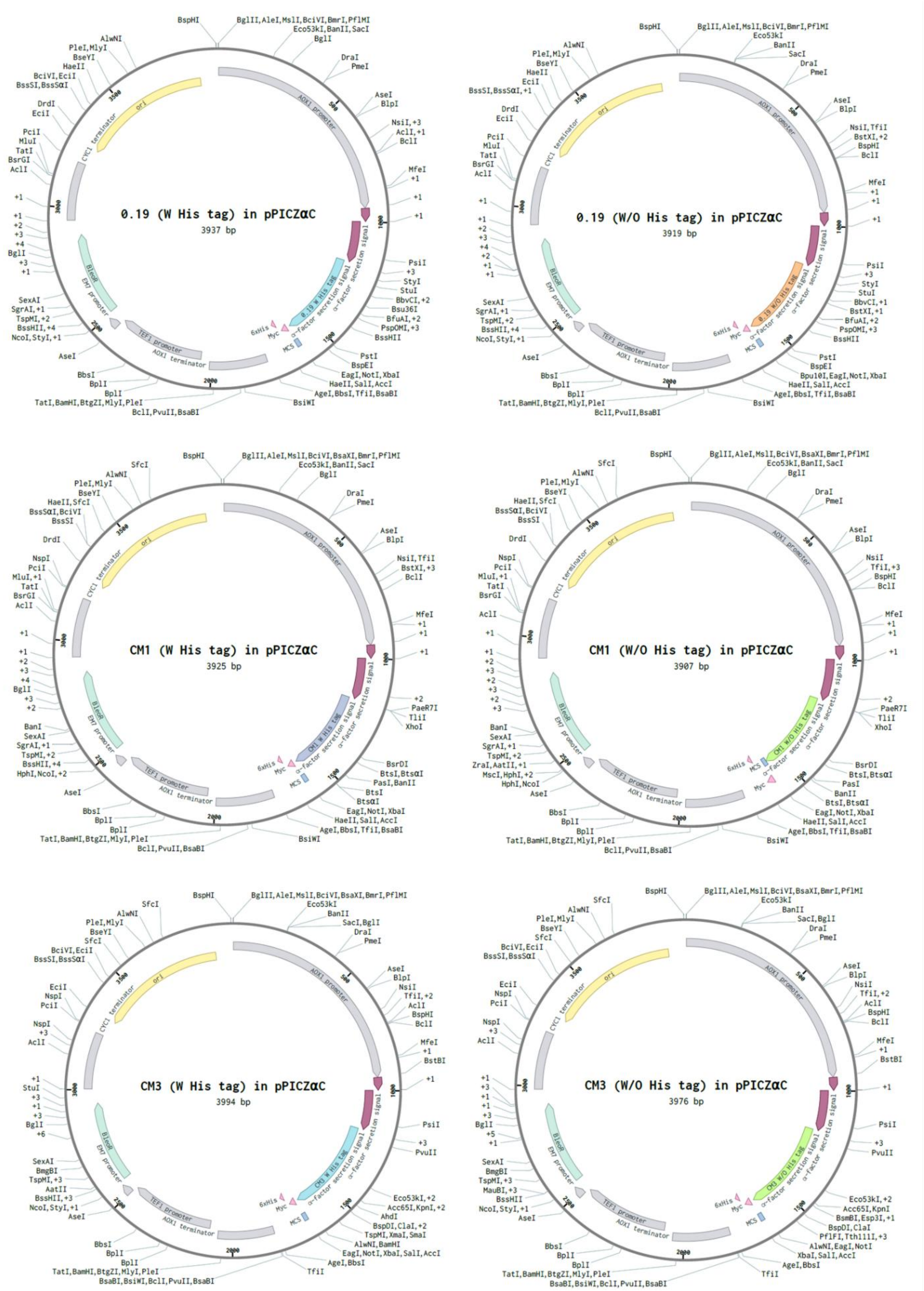

Supplementary Figure S5. Plasmid maps of the pPICZαC (with and without His$_6$ tag) expression vector including amino acid sequences of ATIs 0.19, CM1, and CM3 for heterologous expression in *Pichia pastoris*.

Supplementary Table S1. IgE recognition of individual wheat allergens (nATI, rATIs 0.19, CM1, and CM3) in 94 wheat-sensitized patients. Number of patients with an IgE response >0.1 ISU mL^{-1} (n), their frequency (%), median ($\tilde{x}$), and range of IgE response.

**Allergen**	**Biochemical name**	***n***	**%**	**$\tilde{x}$ (Range)**
nTri a aA_TI	natural ATI preparation	67	71.3	0.62 (0.11 – 63.9)
rATIs	recombinant ATIs	24	25.5	2.6 (0.14 – 46.3)
rTri a 28	dimeric α-amylase inhibitor 0.19	16	17.0	6.4 (0.17 – 46.3)
rTri a 29	tetrameric α-amylase inhibitor CM1/CM2	10	10.6	1.4 (0.14– 5.9)
rTri a 30	tetrameric α-amylase inhibitor CM3	11	11.7	0.9 (0.17 – 6.9)
nTri a aA_TI	natural ATI (ImmunoCAP ISAC E112i)	65 [1]	79.3	1.6 (0.14 – 84.2)

[1] sample size in the ImmunoCAP ISAC E112i array was n=80 as 14 serum samples were depleted after the original microarray test

Supplementary Table S2. IgE recognition of individual wheat allergens (nATI, rATIs 0.19, CM1, and CM3) in 94 wheat-sensitized patients. Number of patients with an IgE response >0.1 ISU mL^{-1} (n), their frequency (%), and median ($\tilde{x}$) in the respective oral food challenge (OFC) groups: OFC−, children phenotyped having a negative OFC result; OFC+, children phenotyped having a positive OFC result. Significance levels correspond to the whole dataset (n=94 and/or n=80).

Allergen	OFC−		OFC+		Significance level
	**n_1 (%)**	**$\tilde{x}$**	**n_2 (%)**	**$\tilde{x}$**	**$p_{(n=94)}$**
nTri a aA_TI	27 (57.5)	0.37	40 (85.1)	0.84	0.005
rTri a 28	6 (12.8)	10.48	10 (21.3)	4.99	0.226
rTri a 29	5 (10.6)	0.75	5 (10.6)	2.51	0.430
rTri a 30	4 (8.5)	1.40	7 (14.9)	0.88	0.799
nTri a aA_TI [1]	26 (61.9)	1.12	39 (97.5)	2.31	<0.001 [2]

[1] natural ATI on ImmunoCAP ISAC E112i array
[2] sample size: n=80
